# Knowledge, Attitude, Motivators, and Barriers to Blood Donation Among Adults in Al-Qunfudah Governorate, Saudi Arabia: A Cross-Sectional Study

**DOI:** 10.7759/cureus.58732

**Published:** 2024-04-22

**Authors:** Safa H Alkalash, Omar A Alturki, Wael S Alzubaidi, Noor M Sabi, Naif A Almarhabi, Mohammed H Alnashri, Bandar M Alsharidi, Atheer O Alothman, Fawaz M Alzubaidi

**Affiliations:** 1 Family Medicine, Menoufia University, Shibin El Kom, EGY; 2 Community Medicine and Health Care, Umm Al-Qura University, Al-Qunfudah, SAU; 3 Medicine and Surgery, Umm Al-Qura University, Al-Qunfudah, SAU; 4 Pediatric Department, King Abdulaziz University, Jeddah, SAU

**Keywords:** knowledge, attitude, blood donation, barriers, motivators

## Abstract

Background: Blood transfusion is one of the most important aspects of managing patients with a variety of medical disorders like thalassemia and sickle cell anemia. Despite this fact, many Saudis hesitate to donate blood and do not know whether blood banks need blood.

Objectives: To evaluate the knowledge, attitude, motivators, and barriers to blood donation among adults in Al-Qunfudah governorate, Saudi Arabia.

Methods: A descriptive cross-sectional study was performed on a convenience sample of 416 adults living in Al-Qunfudah governorate and its dependence, using an online self-administered questionnaire. The obtained data were analyzed statistically using SPSS version 21 (IBM Corp., Armonk, New York, USA).

Results: Among 416 respondents, exactly 232 (55.8%) had overall good knowledge regarding blood donation, and 334 (80.3%) positively perceived it. The best knowledge about blood donation was detected among those aged 21-24 years (p = 0.012), males (p = 0.008), university-educated (p = 0.048), having a government job (p = 0.001), and having a history of donating blood (p = 0.001). The motivators included religious motives (88.2%, n = 367), feelings of self-satisfaction (63.2%, n = 263), and restoring blood circulation (56.7%, n = 236). Barriers to blood donation were fear of being infected (27.6%, n = 115), needle phobia (23.6%, n = 98), fear of general weakness (22.8%, n = 95), didn’t know how to donate (16.8%, n = 70), and fear of seeing blood (13.2%, n = 55).

Conclusion: Although adults in the Al-Qunfudah governorate of Saudi Arabia had positive attitudes toward blood donation, they possessed inadequate knowledge about it. Being younger, male, university-educated, having government jobs, and having a past history of donating blood were factors associated with good knowledge of blood donation. The most common motivators were religious, financial, and maintaining health. However, fear of infection, needle sticks, fear of pain, and hemophobia were the recorded barriers. Public health education is recommended to tackle public concerns regarding blood donation and present its benefits.

## Introduction

Human blood is an essential component of life, and there are currently no substitutes for human blood components [1]. Blood transfusion is most commonly administered and required to save a life, so it is critical that hospitals and urgent care clinics have prompt access to a specified volume of blood and related supplies [2]. Appropriate and safe blood and blood products should be collected and readily available for use in unique medical circumstances to guarantee the best therapeutic outcomes [3].

Surgical operations, trauma, hematological illnesses, and pregnancy-related issues are high utilizers of donated blood. To ensure that hospitals have enough blood for all blood types, voluntary and consistent donations are required. Therefore, blood banks in Saudi Arabia search for donors, test for infectious agents, prepare blood and store it [4].

Every year, over one hundred million units of blood are donated around the world [5]. In Saudi Arabia, blood donation is 13.8 per 1000, which is low in comparison to high-income countries' median blood donation rate of 32.6 per 1000, due to many factors, like poor knowledge [6]. Previous research in Saudi Arabia showed that people's fears regarding agreeing with blood from unknown people or volunteers vary, that half of Saudis surveyed suspected blood donation was hazardous, and that most Saudis weren't aware whether blood banks required blood or not. These findings suggest that a significant portion of Saudi Arabia's population has an unfavorable perception of blood donation and its significance to society [7].

Saudi Arabia is one of the Middle East countries with the highest prevalence of sickle cell anemia and beta-thalassemia, affecting 4.50% and 0.05% of the population, respectively [8]. Sickle cell disease is more prevalent in Al-Qunfudah (135.7 per 1000 Saudis) than in Makkah (30.3) or the Saudi Western region (28.5) [9]. There is a lack of awareness that these conditions are prevalent and require blood transfusions. Therefore, we conducted this study to assess the public's knowledge and attitude, as well as the motivators and barriers to blood donation in Al-Qunfudah Governorate.

## Materials and methods

Study design

A descriptive cross-sectional community-based study was performed through an online questionnaire that was distributed to the general population in Al-Qunfudah governorate through electronic social media such as Telegram, What’s App, and Twitter. This survey looked at the knowledge, attitudes, motivators, and barriers to blood donation among adults in Al-Qunfudah governorate.

Study period

The research data were collected over a period of three months, from January to March 2023.

Study population

Inclusion criteria: Saudi and non-Saudi adults of both genders who were living in Al-Qunfudah governorate and its dependence.

Sample size

The Epi-Info software (CDC, Atlanta, Georgia, USA) estimated the minimum required sample size. It was 384 based on the total number of adults in Al-Qunfudah governorate (300,516) and the frequency of adequate knowledge about blood donation (60.2%) [10], at a confidence interval of 95%.

Data collection tool

The study researchers designed the survey for data collection after a literature review [10,11] and group discussion to select the relevant questions. After creating the survey, it was rechecked by a panel of three experts in family medicine, hematology, and internal medicine who approved its validity. In December 2022, the survey link was disseminated on different electronic platforms like Telegram and WhatsApp until it obtained 35 responses as a pilot study to assess the study methodology and pretest the designed survey. The data from the pilot was analyzed to examine its items’ reliability through Cronbach’s alpha. The value of Cronbach's alpha coefficient for the full questionnaire was 0.83, indicating its reliability [12]. All the questionnaire items were completed by the respondents; therefore, there is no need for any item alteration. Responses gathered in the pilot study were omitted from the main data analysis.

The survey consisted of 32 items that were subdivided into four main domains. The first domain involved seven items that inquired about the respondents’ demographic data, like age, gender, residence, education, and occupation, in addition to their history of blood transfusion. The next part involved 18 questions about their knowledge of blood donation, such as whether they knew their blood groups, the site of blood donation, and whether smokers, pregnant women, and anemic patients could donate or not. The third part involved five items that evaluated their attitudes towards blood donation. The last portion of the survey involved two items that inquired about motivators and barriers to blood donation.

Scoring System of Knowledge and Attitude Toward Blood Donation

Each incorrect or do not know answer received a score of zero, while the correct response scored one. The overall knowledge level regarding blood donation was assessed by summing up discrete scores for different correct knowledge items. The mean number of correct answers provided by the participants was 12. The overall knowledge score was categorized as good when the participant correctly answered 12 items or more. The same was applied to the attitude score; the mean attitude score was three; those who correctly answered three out of five answers or more were considered to have a positive attitude.

In January 2023, the whole public was given access to the poll via the Whatsapp, Telegram, Twitter (X Corp., San Francisco, CA, USA), and Snapchat.

Ethical approval considerations

This study was approved by the bioethical committee of Umm Al-Qura University, Makkah City, KSA, in September 2022 with an ethical approval number (HAPO-02-K-012-2022-09-1170). All participants were volunteers, and their consents were obtained before starting data collection through an introductory question. Data confidentiality was maintained throughout the study course.

Data analysis

Statistical Package for Social Sciences version 21 (IBM Corp., Armonk, New York, USA) was used to process and analyze the data after it had been gathered. With an alpha threshold of 0.05, all statistical methods were two-tailed, and a p-value of less than or equal to 0.05 was considered significant. Descriptive analysis was done by prescribing frequency distribution and percentage for study variables, including adults' personal data, education, blood donation experience, and causes. Also, knowledge regarding blood donation and respondents’ attitudes were tabulated, while overall knowledge, motives for donation, and barriers against donation were graphed. A cross-tabulation to show factors associated with the study adults' knowledge of blood donation was carried out with a Pearson chi-squared test for significance and an exact probability test if there were small frequency distributions.

## Results

A total of 416 eligible adults completed the study questionnaire. Participants ages ranged from 18 to 62 years, with a mean age of 29.1 ± 10.7 years old. The majority of 249 (59.9%) participants were males. Most of them (86.3%, n = 359) were university graduates. Nearly half of the sample (41.8%, n = 174) were students. A total of 243 (58.4%) were singles. Regarding blood donation experience, it was reported among 188 (45.2%) respondents. The main reasons for blood donation included religious reasons (53.7%, n = 101), for relatives and friends (25%, n = 47), for driving licenses (24.5%, n = 46), and it was a regular habit among 44 (23.4%) respondents (Table 1).

**Table 1:** Personal characteristics of study participants in Al-Qunfudah governorate, Saudi Arabia.

Personal data	N	%
Age in years		
18-20	75	18.0%
21-24	143	34.4%
25-30	60	14.4%
31-40	63	15.2%
41- 50	57	13.7%
51-62	18	4.3%
Gender		
Male	249	59.9%
Female	167	40.1%
Educational level		
Below secondary	12	2.9%
Secondary school	45	10.8%
University graduate	359	86.3%
Employment		
Unemployed/retired	82	19.7%
Student	174	41.8%
Governmental sector	109	26.2%
Private sector	39	9.4%
Military sector	12	2.9%
Marital status		
Single	243	58.4%
Married	165	39.7%
Divorced/widow	8	1.9%
Do you have any experience donating blood?		
Yes	188	45.2%
No	228	54.8%
The reason for previous blood donation (n = 188)		
For religious cause	101	53.7%
Relative and friends	47	25.0%
For driving license	46	24.5%
I donate regularly	44	23.4%
For strangers from social media	6	3.2%

A total of 377 adults, representing 90.6% of the study sample, knew that donating blood from the arm is the most common route; 90.1% (n = 375) understood that donating blood improves the body's blood circulation; 82.5% (n = 343) recognized the necessity of doing laboratory tests on blood donors prior to donation; and 76% (n = 316) knew their blood grouping. Sixty-six responders (15.9%) reported that blood donation can cause anemia in the donors; 52.2% (n = 217) said that smokers can donate blood; and 39.2% (n = 163) of the sample identified that one blood bag can save three lives (Table 2).

**Table 2:** Blood donation knowledge among the study sample in Al-Qunfudah governorate, Saudi Arabia.

Knowledge items	Correct		Incorrect		I don't know	
	N	%	N	%	N	%
You know the blood group you belong to	316	76.0%	23	5.5%	77	18.5%
Donating blood from the arm is the most common method	377	90.6%	1	0.2%	38	9.1%
People can donate to others who have the same blood type	308	74.0%	58	13.9%	50	12.0%
A person who is infected with Hepatitis C can donate blood	10	2.4%	259	62.3%	147	35.3%
Anemics can donate blood	10	2.4%	331	79.6%	75	18.0%
It is fundamental to do laboratory tests on blood donors	343	82.5%	20	4.8%	53	12.7%
Anemia can be caused by blood donation	66	15.9%	224	53.8%	126	30.3%
Blood donors can donate every three months	260	62.5%	39	9.4%	117	28.1%
Donations should only be made for relatives and family members	13	3.1%	390	93.8%	13	3.1%
Donating blood improves the body's blood circulation	375	90.1%	0	0.0%	41	9.9%
Donating blood stimulates the bone marrow to produce new blood cells	310	74.5%	5	1.2%	101	24.3%
Loses of body weight approximately 650 calories per pint of blood	126	30.3%	30	7.2%	260	62.5%
Pregnant can donate	21	5.0%	205	49.3%	190	45.7%
Donation lowers the risk of cardiovascular diseases	238	57.2%	8	1.9%	170	40.9%
One bag of blood can save three lives	163	39.2%	25	6.0%	228	54.8%
Donor must be between 18 and 65 years	271	65.1%	18	4.3%	127	30.5%
Hemoglobin levels should be 14-17 g/dl for men and 12-14 g/dl for women	176	42.3%	15	3.6%	225	54.1%
Smokers can donate blood	217	52.2%	72	17.3%	127	30.5%

**Figure 1:**
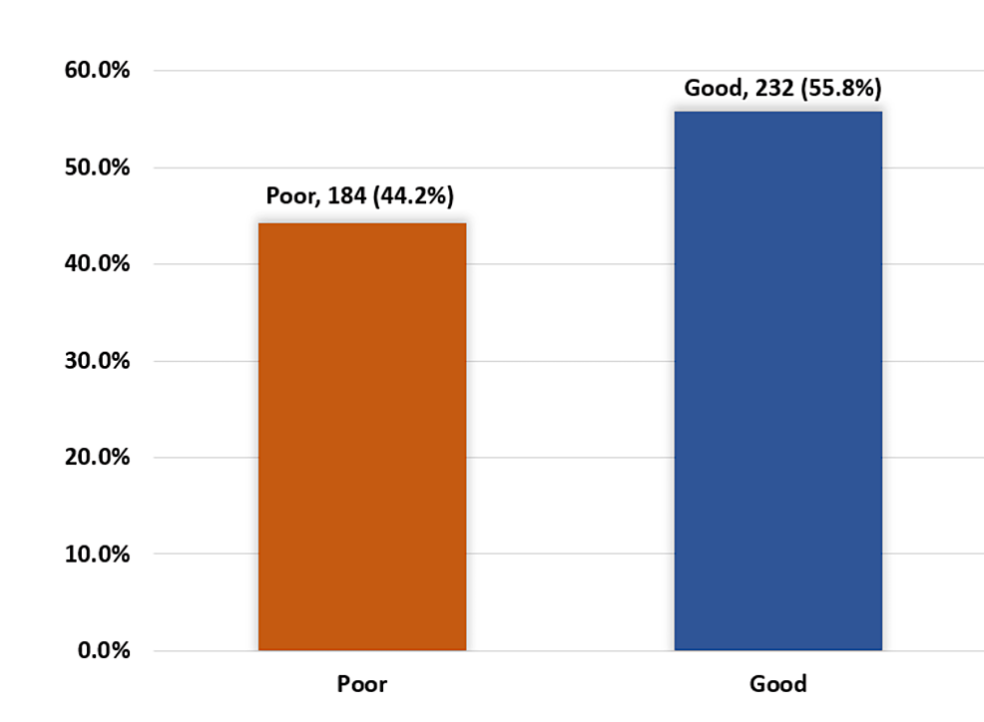
Overall knowledge level regarding blood donation among study adults in Al-Qunfudah governorate, Saudi Arabia.

Exactly 232 (55.8%) of the study adults had an overall good level of knowledge regarding blood donation, while 184 (44.2%) had poor knowledge (Figure 1).

A total of 398 (95.7%) thought that it was a responsibility to donate blood to save lives. Of the study sample, 383 (92.1%) agreed that others' donations motivate them, 303 (72.8%) acknowledged the importance of blood donation, and 236 (56.7%) believed that people donate blood for religious reasons. Only 2.9% reported they would make a monetary donation. Finally, 334 (80.3%) of the study subjects positively perceived blood donation (Table 3).

**Table 3:** Adults' attitudes regarding blood donation, Al-Qunfudah governorate, Saudi Arabia.

Attitude item	Agree		Disagree		Neutral	
	N	%	N	%	N	%
It is essential to donate blood	303	72.8%	41	9.9%	72	17.3%
Others are motivated to donate blood because of your donation	383	92.1%	6	1.4%	27	6.5%
You will make a monetary donation	12	2.9%	354	85.1%	50	12.0%
People donate blood out of religious motives	236	56.7%	74	17.8%	106	25.5%
It is a responsibility to donate blood to save lives	398	95.7%	5	1.2%	13	3.1%

A total of 87 (60.8%) of the study subjects aged 21-24 had a better knowledge level than 29 (38.7%) of others aged less than 20 years, with recorded statistical significance (p = 0.012). Also, 152 (61%) of male adults had better knowledge compared to 97 (47.9%) of females (p = 0.008). Likewise, good knowledge was detected among 204 (56.8%) of those with university graduation, compared to 3 (25%) of those with a lower level of education (p = 0.048). Good knowledge was identified among 78 (71.6%) of adults in the governmental sector compared to 31 (37.8%) of unemployed respondents (p = 0.001). Those who had experience donating blood showed the best knowledge, representing 68.1% (n = 128) with p = 0.001 (Table 4).

**Table 4:** Factors related to the study participants' knowledge of blood donation. P: Pearson X^2^ test, $: exact probability test, *P < 0.05 (significant).

Factors	Overall knowledge level				p-value
	Poor		Good		
	N	%	N	%	
Age in years					0.012*
18-20	46	61.3%	29	38.7%	
21-24	56	39.2%	87	60.8%	
25-30	25	41.7%	35	58.3%	
>30	57	41.3%	81	58.7%	
Gender					0.008*
Male	97	39.0%	152	61.0%	
Female	87	52.1%	80	47.9%	
Educational level					0.048*^$^
Below secondary	9	75.0%	3	25.0%	
Secondary school	20	44.4%	25	55.6%	
University graduate	155	43.2%	204	56.8%	
Employment					0.001*
Unemployed/retired	51	62.2%	31	37.8%	
Student	76	43.7%	98	56.3%	
Governmental sector	31	28.4%	78	71.6%	
Private sector	19	48.7%	20	51.3%	
Military sector	7	58.3%	5	41.7%	
Marital status					0.135^$^
Single	115	47.3%	128	52.7%	
Married	64	38.8%	101	61.2%	
Divorced/widow	5	62.5%	3	37.5%	
Do you have any experience donating blood?					0.001*
Yes	60	31.9%	128	68.1%	
No	124	54.4%	104	45.6%	

**Figure 2:**
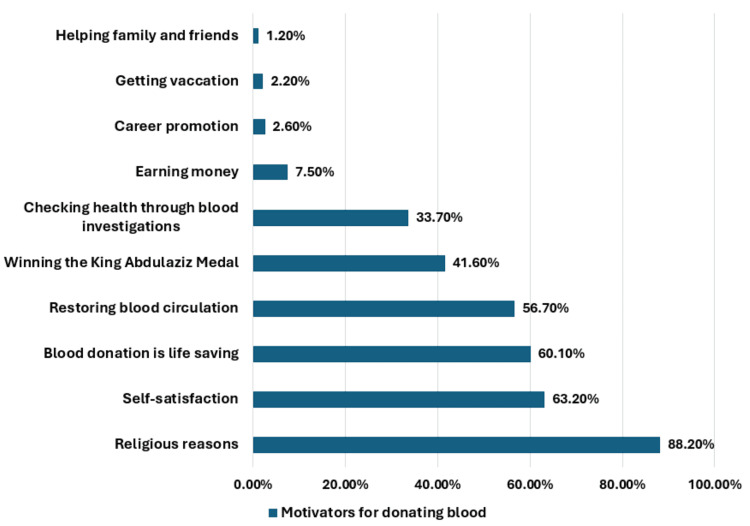
The participants' driving motives behind donating blood.

The most recorded motivators for blood donation were religious motives (88.2%, n = 367), feelings of self-satisfaction (63.2%, n = 263), recognizing the importance of donating blood (60.1%, n = 250), restoring blood circulation (56.7%, n = 236), donating to get the King Abdulaziz Medal (41.6%, n = 173), and checking health with a laboratory test (33.7%, n = 140) (Figure 2).

**Figure 3:**
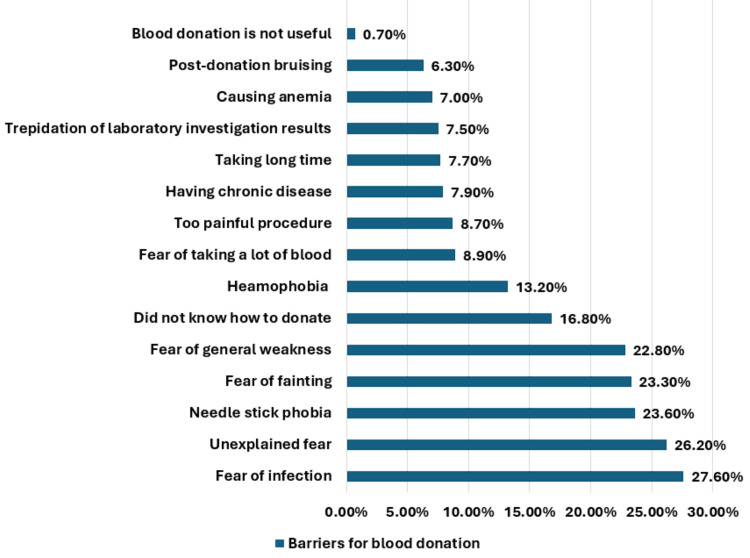
The barriers among the participants in blood donation.

The major barriers to blood were fear of getting infected due to inappropriate sterilization and infection control measures (27.6%, n = 115), fear of an unknown cause (26.2%, n = 109), needle phobia (23.6%, n = 98), fear of fainting (23.3%, n = 97), fear of general weakness (22.8%, n = 95), not knowing how to donate (16.8%, n = 70), and hemophobia (13.2%, n = 55) (Figure 3).

## Discussion

Globally, there is a growing need for blood, yet getting safe blood is still a problem, and every nation should have a national healthcare policy and infrastructure to maintain the provision of safe and sufficient blood for those in need [13]. In 2020, wide-scale research was done in Saudi Arabia concerning blood donation. It was reported that the Al-Qunfudah Governorate has five blood banks related to the Ministry of Health, and the total annual blood donors was 9906 out of 220000 people, which is considered low [14].

As blood donation is a voluntary work that depends on volunteers’ knowledge and attitude towards it and can be affected by a number of motivators and challenges, this study sought to ascertain the knowledge and attitudes of adults in the Governorate of Al-Qunfudah, Saudi Arabia, about blood donation, as well as their perspectives on the factors that encourage and hinder blood donation. Out of the 416 adults who took part in our poll, 188 (45.2%) donated blood. This is like that in a previous Saudi study in Jeddah [15], but it is much greater than in a study in Qatar [16] and Ethiopia [17], where only 15% and 22%, respectively, of their samples had a history of blood donation. The variation in results may be due to differences in study settings and the characteristics of each study sample.

Regarding knowledge of blood donation among adults from Al-Qunfudah Governorate, approximately 55.8% of the study subjects had adequate knowledge of blood donation, as most of them demonstrated a solid understanding of its importance as well as the necessary precautions and potential complications involved. These findings align with similar studies [1,3,15]. Knowledge of blood donation was found to be better in those ages 21 and 24 (p = 0.012), males (p = 0.008), university graduates (p = 0.048), and those with government jobs (p = 0.001). The same was reported by Alfouzan in her Saudi study [15] and Majdabadi et al. in Iran [3]. In this study, people who previously donated blood exhibited better knowledge than the others (p = 0.001). This is supported by other studies in Iran and Qatar [3,16].

The study reflects the positive attitudes of the respondents regarding the importance of blood donation (72.8%), unpaid donation (85.1%), feeling of responsibility to save other's lives through donation (95.7%), and encouraging others to donate (92.1%). This positive trend in blood donation is a reason for optimism. Similar findings were reported by Melku and his colleagues in Ethiopia [1], Alfouzan in Saudi Arabia [15], and Urgesa et al. in Ethiopia [17]. Most of their studies’ subjects perceived voluntary, unpaid blood donation as good work. However, a study in Iran revealed negative attitudes toward blood donation among university students [3]. The differences may be related to cultural variance.

Motivators for blood transfusion were religious advantages (88.2%), feelings of self-satisfaction (63.2%), recognizing the importance of donating blood (60.1%), restoring blood circulation (56.7%), donating to get the King Abdulaziz Medal (41.6%), and checking health with a laboratory test (33.7%). As Saudi Arabia is an Islamic country, it is expected that most Muslims help others everywhere and donate blood for needy people to gain religious benefits from Allah. Previously done Saudi studies found that more than 70% of their samples were motivated to donate blood due to religious reasons [15,18]. France and his colleagues reported that role modeling and altruism were the motivators for blood donation [19].

In this study, the recorded barriers to blood donation were fear of many issues like poor sterilization and infection transmission (27.6%), unknown cause of fear (26.2%, n = 109), needle phobia (23.6%), fainting (23.3%), general weakness (22.8%), sight of blood (13.2%), and don’t know how to donate (16.8%). Many of these barriers were reported by different researchers in different countries [7,15,19-21]. To overcome such barriers, people should receive more information about the process of blood donation and ensure that it is done under complete aseptic conditions by a well-trained team. 

Limitations and strengths

There are certain limitations in the study that should be taken into account. Initially, the study participants were chosen as a convenience sample, which could cause the target sample to be maldistributed. However, this could be overcome in the future by employing a simple random sampling technique. The second constraint derived from the virtual method of data collection, which was limited to educated individuals and those having internet access. Notwithstanding the previous drawbacks, this survey represented the public's knowledge and attitudes about blood donation for the first time in the Governorate of Al-Qunfudah.

## Conclusions

This study highlighted the insufficient level of public knowledge of blood donation despite the positive attitudes toward it in the Al-Qunfudah Governorate of Saudi Arabia. The characteristics of having government positions, being younger, male, educated at university, and having donated blood in the past were linked to having strong blood donation knowledge. The three main driving forces were maintaining one's health, finances, and religion. The known obstacles, however, were hemophobia, fear of needle sticks, fear of infection, and fear of fainting. There is an urgent need for focused educational interventions to improve public comprehension and correct misconceptions regarding blood donation. The recorded motivators and barriers may be used to encourage and facilitate blood donation in this region.
